# Patient and Public Involvement in Occupational Therapy Health Research: A Scoping Review

**DOI:** 10.1177/15394492221096058

**Published:** 2022-05-13

**Authors:** Toril Beate Røssvoll, Tove Aminda Hanssen, Jan H. Rosenvinge, Kristin Liabo, Gunn Pettersen

**Affiliations:** 1 UiT The Arctic University of Norway, Tromsø, Norway; 2 University of Exeter Medical School, UK

**Keywords:** occupational therapy, scoping reviews, participation, engagement

## Abstract

Patient and public involvement (PPI) in research has the potential to improve research validity and relevance. Objectives: To explore how PPI has been carried out and how its impacts have been reported in occupational therapy (OT) health research. Methodology: Scoping review based on a search in four databases for OT research with descriptions of PPI, published between 2010 and 2020. Results: Across the 17 included studies PPI was reported in all stages of research. Descriptions of how PPI was carried out varied across the studies, and details with respect to the kind of approach used were lacking. Positive impacts on research design, research ethics, public collaborators and researchers were reported, but only anecdotally. Reflections and challenges related to PPI were also addressed. Implications: In future studies, comprehensiveness and consistency is needed to document the diversity of how PPI is carried out in OT health research.

Patient and public involvement (PPI) entails active collaboration between researchers and either patients or members of the public in all or some parts of the research process (Hayes et al., 2012; Sacristán et al., 2016). PPI has potential to enhance the relevance and impact of health research (Hayes et al., 2012), is increasingly considered to be an essential part of the whole research process (de Iongh et al., 2021), and has become a prerequisite to consider for research funding. The evidence base continues to grow on the impact PPI can have on health research. When researchers work directly with patients or members of the public, they might gain new understandings of what the relevant and important research issues and questions are and how to prioritize them to benefit patients (Hayes et al., 2012).

Several PPI reviews offer suggestions on creating a scientific framework for the PPI process (Forsythe et al., 2019; Shippee et al., 2013). Oliver and colleagues (Oliver et al., 2015) present a framework emphasizing both PPI activities and tools for evaluation. However, PPI frameworks have been in little use beyond the groups that developed them (Greenhalgh et al., 2019).

There has been a shift in focus from convincing researchers *why* they need to involve public collaborators to *how* to do it (de Iongh et al., 2021). “How to do PPI” is of special interest for occupational therapy (OT) research for many reasons. Involvement principles, such as inclusion and working together as equal partners, aligns with the professional philosophy underpinning OT (Harries et al., 2020). The overall intention of OT research is to understand the place of occupation within and throughout people`s lives, in communities, and society at large (Nayar & Stanley, 2015). Participation in terms of involvement or sharing, particularly in an activity, is a fundamental aspect of OT (Law, 2002). Involvement of patients and members of the public in research has been coined as an essential part of client-centered practice in OT (Hammell et al., 2012).

In addition to requests for a stronger focus on PPI in OT research (Asaba & Suarez-Balcazar, 2018; Haywood et al., 2019), there have been calls to increase the sharing of how involvement is approached and experienced (de Iongh et al., 2021; Gustafsson et al., 2019). However, we are aware of only one previous review in OT research, limited to publications in the Australian *Occupational Therapy Journal* (Cox et al., 2021). Although OT research articles are included in PPI reviews in research on disability (Joss et al., 2016) and rehabilitation (Camden et al., 2015), little is known about how PPI has been undertaken in OT research specifically. A scoping review can guide the process of exploring and mapping the body of existing literature (Arksey & O’Malley, 2005), and fill this gap of knowledge. To summarize a broad range of literature can also assist in applying research findings to research practice (McKinstry et al., 2014).

With the aim of mapping PPI in OT health research from a wide range of journals, we conducted a scoping review to explore (1) how PPI in OT health research has been carried out and (2) what kind of impacts from PPI have been reported.

## Methods

### Overview

The outline for the present scoping review adhered to the Arksey and O’Malley (2005) framework and the advancements of the methodology (Levac et al., 2010; Peters et al., 2020). The research protocol was registered at Open Science Framework (Røssvoll & Pettersen, 2020).

In this study, PPI was defined as involvement in research being carried out “with” or “by” members of the public rather than “to,” “about” or “for” them (Hayes et al., 2012). For brevity, we use the term “public collaborator” when referring to service user researchers, or patients and members of the public involved in research. By the term “impact,” we refer to any changes made from PPI input in the studies identified (Staley, 2009).

### Identification of Relevant Studies

The search strategy was based on a published PPI search filter (Rogers et al., 2017) combined with topical search terms to detect studies within OT. The search filter was developed and tested for MEDLINE (Rogers et al., 2017) and adopted to the other databases chosen for this review. A senior librarian assisted in developing and validating the search strategy. Search terms included a variation of index-terms and text words, such as consumer participation, patient participation, OT, and occupational science. Supplemental material 1 displays the complete search strategy for Medline (Ovid), CINAHL (EBSCOhost), Embase (Ovid), and Scopus (Elsevier). Back and forward citation tracking of the included articles was performed in the Web of Science.

### Study Selection

The inclusion criteria were original OT health research published from January 2010 to August 2020 in English, and with a description of PPI, or similar terms reflected in our search strategy, in the methods section. The time frame aimed to capture the increased focus on PPI in research during this decade (de Iongh et al., 2021; Oliver et al., 2015; Shippee et al., 2013). Excluded studies were nonacademic publications, congress papers, opinion papers, editorials, and multiprofessional studies as well as case reports, and studies focusing on OT students, OT educators, or solely OT clinicians.

**Figure 1.**
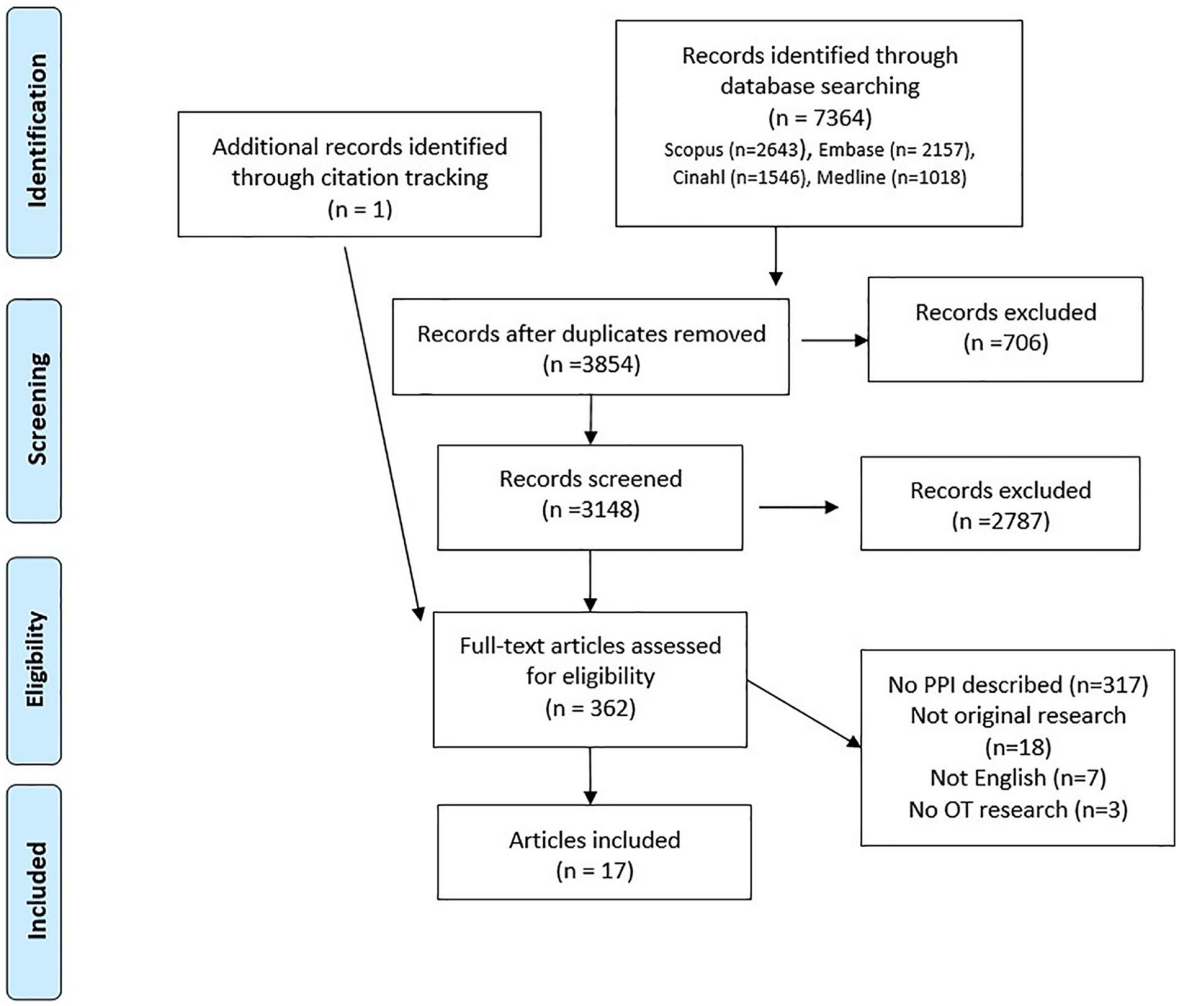
PRISMA flow diagram for study selection (Moher et al., 2009).

To identify eligible studies, titles, and abstracts were independently screened by two of the authors, and full-text eligibility was assessed by first author. Of these, 10% were scrutinized for validation purposes by the last author. The study-selection procedure is displayed in Figure 1 using a Preferred Reporting Items for Systematic Reviews and Meta-Analyses (PRISMA) flow diagram (Moher et al., 2009).

### Data Charting, Collating, and Summarizing the Result

A data charting form was developed to display study characteristics, the PPI terms used, as well as descriptions of how PPI is carried out and impact of PPI (see supplemental material 2). We chose involvement related to research phases as one way to describe how PPI was carried out, besides PPI approach and role of public collaborators. Analysis of impact was displayed within the INVOLVE framework, which outlines nine different kinds of impact of PPI; research agenda, research design and delivery, research ethics, public advisors, researchers, research participants, the wider community, and the implementation or change resulting from the research in which people were involved, besides factors influencing whether involvement makes a difference (Staley, 2009).

### PPI in This Scoping Review

To enhance the relevance and quality of this research, three public collaborators were invited to join the research group. The public collaborators’ role was to comment on the research with views anchored in their experiences of being involved in research as patients or relatives of patients. They also draw on their knowledge of using services, for example receiving OT. The public collaborators engaged in conversations at research group meetings, and provided input concerning the review question, helped to refine the protocol, including the search strategy and data extraction approach. Notable impact from the public collaborators included refining the terminology used in this review and avoiding the commonly used term “lay” member.

## Results

In total, 17 studies from 2010 to 2020 were included, 14 with a qualitative design, the remaining three with a mixture of quantitative and qualitative design. Most studies were conducted in the United Kingdom (7), Canada (4), and Australia (3). Across the included studies, PPI was reported in all stages of research, but only one study (Crabtree, Ohm, et al., 2016) reported involvement in all stages.

Nine studies integrated PPI in the description of research approach, described as “photovoice with user involvement as its core” (Andonian & MacRae, 2011; Birken & Bryant, 2019), “participatory action research” (Baker & Procter, 2014; Crabtree, Ohm, et al., 2016; Trentham & Neysmith, 2018), “a method for involving stakeholders in a structured process” (Nielsen et al., 2019), or “participatory research using a qualitative approach” (Makdisi et al., 2013; Ripat et al., 2010).

Two studies emphasized the congruence between the participatory approach in research to occupational justice approach. The congruence was argued by the seeking of perspectives of occupation through marginalized eyes and using occupation as a means for expression, engagement, and collaboration (Birken & Harper, 2017), and how the inclusive approach generated opportunities to make choices about involvement in specific research tasks and activities (Bryant et al., 2016).

Nine of the studies reported PPI at the early stages of research process, in discussions about study focus and design, while 13 studies reported PPI in data collection and 14 in analysis. Nine studies reported PPI in latest stage of research, for example, dissemination; however, across the included studies PPI was reported in all stages of research.

None of the studies reported on a formal evaluation of impact, but 11 reported on impact of PPI anecdotally, mainly in relation to impact on research design. Only five studies reported impacts on researchers and public collaborators. Factors influencing the impact of involvement were considered by four of the included studies.

Study design, role of public collaborators, involvement related to research phases, co-authorship and reporting of impact in the included studies are displayed in Table 1. Of note is the increased number of studies the past 5 years, the diversity of PPI approach and the different roles held by public collaborators.

**Table 1.** Characteristics of the Included Studies.

About the included studies		Public collaborators			Involvement in research phases						Co-authors	Impact of PPI
First author, year, country of origin	Design	Also study participant	With lived experience	Other^a^	Planning	Developing research tools	Recruitment	Data collection	Analysis	Dissemination	x = yes	Described (x = yes)
Crabtree, Ohm et al. (2016), USA	Qualitative	x	x		x	x	x	x	x	x	x	x
Birken & Bryant (2019), UK	Qualitative	x	x		x		x	x	x	x		x
Bryant et al. (2016), UK	Qualitative		x		x	x	x	x	x			x
Ripat et al. (2010), Canada	Qualitative		x		x		x	x	x	x		x
Turcotte et al. (2019), Canada	Qualitative		x		x		x	x	x	x		x
Birken & Harper (2017), UK	Qualitative		x			x		x	x	x	x	x
Craik et al. (2010), UK	Qualitative			x		x	x		x	x		
Makdisi et al. (2013), UK	Qualitative		x		x			x	x	x	x	x
Andonian & McRae (2011), USA	Qualitative	x	x			x		x	x			
Ball & Shanks (2012), UK	Mixed methods		x			x		x	x			
Simpson et al. (2020), UK	Qualitative			x	x				x	x		
Williamson & Ennals (2020), Australia	Qualitative			x	x	x	x					x
Baker & Procter (2014), Australia	Qualitative	x	x		x			x				
Nielsen et al. (2019), Denmark	Mixed methods	x	x					x	x			x
Restall et al. (2017), Canada	Qualitative		x						x	x	x	x
Trentham & Neysmith (2018), Canada	Qualitative	x	x					x	x			x
Hancock et al. (2015), Australia	Mixed methods		x					x				

*Note.* PPI = patient and public involvement.

a Studies where public collaborators are not people with lived experience relevant to the study, but “people who promoted the interests and concerns of service users,” “youth and family care advisors,” and “former carers.”

## How PPI Was Carried Out

Reflecting the wide range of focus and variation in study populations across the included studies, they also differed in who were invited as public collaborators. In seven studies, individuals with experience of living with mental illness, or service user experience within mental health were the public collaborators (Baker & Procter, 2014; Birken & Bryant, 2017, 2019; Bryant et al., 2016; Hancock et al., 2015; Makdisi et al., 2013; Williamson & Ennals, 2020). Other public collaborators included older adults (Andonian & MacRae, 2011), residents in prison (Crabtree, Ohm, et al., 2016), or persons living with chronic conditions (Nielsen et al., 2019; Restall et al., 2017). Public collaborators did not always have patient experience relevant to the study. For example, former carers of people living with motor neurone disease were public collaborators instead of patients themselves (Simpson et al., 2020) and family care advisors were public collaborators in a study on young people engaged in community mental health services (Williamson & Ennals, 2020).

Descriptions of how PPI were carried out varied considerably across the studies. PPI at the initial stages of research was for example described as involvement in processes related to funding and study approvals (Birken & Bryant, 2019), development of research ideas or aims (Crabtree, Ohm, et al., 2016; Turcotte et al., 2019), concept or study design (Simpson et al., 2020). PPI was also reported with respect to involvement in the data collection strategy (Bryant et al., 2016; Makdisi et al., 2013): by including public collaborators in interviewer team (Birken & Harper, 2017; Crabtree, Ohm, et al., 2016; Hancock et al., 2015) or in the collection of data (Birken & Bryant, 2019; Turcotte et al., 2019). Involvement in data analysis was reported as, for example, involving public collaborators in interpreting the findings (Birken & Harper, 2017), reviewing the coding scheme (Restall et al., 2017), identifying categories (Andonian & MacRae, 2011; Crabtree, Ohm, et al., 2016), and regular discussions during data analysis (Bryant et al., 2016; Makdisi et al., 2013). Involvement in dissemination of results included help to formulate recommendations (Ball & Shanks, 2012), involvement in education session (Ripat et al., 2010) or simply as “public collaborators were involved in the dissemination” (Simpson et al., 2020; Turcotte et al., 2019), without further descriptions. In four studies, public collaborators were co-authors of the research article (Birken & Harper, 2017; Crabtree, Ohm, et al., 2016; Makdisi et al., 2013; Restall et al., 2017).

The PPI approach included “service user advisory panel” (Ball & Shanks, 2012), “steering group with diverse members including service users” (Bryant et al., 2016), “user forum” (Craik et al., 2010), “involving various stakeholders in a structured process” (Nielsen et al., 2019), or as “research team with diverse members” including public collaborators (Bryant et al., 2016; Makdisi et al., 2013; Restall et al., 2017). One study established an advisory group including service users, with the rationale to oversee the research and ensure quality (Birken & Bryant, 2019). Examples of the composition of teams included students and clinicians (Bryant et al., 2016; Makdisi et al., 2013) or various stakeholders as clinicians and representatives from related organizations (Ripat et al., 2010).

### Impact of PPI in Research

PPI impact on research design linked to stages in research. Public collaborators had impacted on the data collection tools, by including questions considered important to the study population (Bryant et al., 2016; Nielsen et al., 2019; Williamson & Ennals, 2020). Impact on data collection was described as an advantage related to becoming familiar with the study topic (Birken & Harper, 2017; Crabtree, Ohm, et al., 2016), and by helping participants to express themselves more freely, gaining a deeper understanding (Bryant et al., 2016).

Public collaborators experienced impacts from being involved, including personal development and enhanced knowledge of the research topic (Ripat et al., 2010). Enjoyment and satisfaction were reported as mutually beneficial for public collaborators and researchers (Turcotte et al., 2019). Two of the studies reported that working with public collaborators enabled researchers to approach their research topic in a more rigorous and accessible way (Bryant et al., 2016), and they reported being sensitized to the context of their study (Makdisi et al., 2013). Frequent team discussions and involvement in analyses reduced the researchers’ position of power in the relation to the participants (Turcotte et al., 2019). The enablement of power sharing is reported through development of respectful relationships, the enabling of public collaborators to set the research direction, and engaging in collaborative decision-making processes (Ripat et al., 2010).

One study described impact on research ethics related to a rigorous and accessible approach (Bryant et al., 2016). Another study reported how involvement provided detailed knowledge and understanding of the context of the study topic and ensured a practical focus when discussing ethical issues (Makdisi et al., 2013).

Five studies presented reflections and challenges related to PPI. Change in organization and membership status of public collaborators limited involvement throughout the research project, in particular by precluding involvement in dissemination (Trentham & Neysmith, 2018). Involvement throughout the research project was challenged when the availability of public collaborators was difficult to predict (Crabtree, Ohm, et al., 2016). The importance of role agreement was emphasized, especially where various people were involved across the research process (Bryant et al., 2016). Training and provision of accessible information for the people involved were described, but not how it influenced the involvement of public collaborators (Ball & Shanks, 2012).

## Discussion

This scoping review aimed to map PPI in OT health research by exploring how PPI has been carried out, and what kind of impacts from PPI that have been reported. The overall findings from the 17 included studies affirm the varied nature of PPI in OT health research and reflect the breadth of the OT research field. PPI was reported in all stages of research across the included studies. Positive, yet anecdotally reported impacts on research design, research ethics, public collaborators, and researchers were provided along with reflections and challenges related to PPI.

The reporting of positive impacts of PPI in research aligns with a previous systematic review stating that PPI has a positive impact on research, enhancing the quality of research and ensuring its appropriateness and relevance (Brett et al., 2014). Moreover, the likelihood of a positive impact increases by arranging for involvement throughout an entire research project, rather than just at some stages (Staley, 2009). However, the academic research culture as well as the traditional style of reporting results tend to focus on positive findings (Staley, 2009). We found no reporting of directly negative impact of PPI, yet reflections and challenges related to involvement throughout the research project were presented.

Involvement of public collaborators in deciding a study’s focus and design concurs with the fundamental argument for PPI to ensure that research is relevant and addresses the interest and need of end users of research (Joss et al., 2016; Slattery et al., 2020; Staley, 2009). The diversity of how PPI was carried out across the included studies resonated well with PPI reviews in other areas of health research (Miah et al., 2019; Slattery et al., 2020) in the sense that diversity mirrors various research aims, designs, research contexts including the researchers and public collaborators competence and characteristics, and the resources available.

From an occupational perspective, PPI in research requires an inclusive and broad understanding of the nature of participation as encompassing the cultures and people involved (Bryant et al., 2011). Participation is a central aspect within OT (Law, 2002) and to enable participation is a core task for occupational therapists. This can be an advantage for OT researchers when initiating and facilitating PPI in research. PPI is a complex, social process, consisting of elements interacting in a dynamic relationship (Bryant et al., 2011; Staley, 2009). The occupations in a research process can be designed and adapted by the researchers to enable the public collaborators to participate in diverse ways (Bryant et al., 2011), identified as factors influencing the impact of involvement (Staley, 2009).

Two of the included studies published separately about PPI (Bryant et al., 2019; Crabtree, Wall, et al., 2016), while the remaining studies included a short description of their PPI in their main findings paper. Structural challenges to reporting PPI in research papers include word limitations and no dedicated PPI sections. Considering PPI being an emerging field, separate papers with room for comprehensive descriptions can have a value.

### Methodological Considerations

Using a published search filter (Rogers et al., 2017) is a strength of this review, probably resulting in an increment of validity by finding more relevant studies initially, compared to other PPI reviews (Camden et al., 2015; Cox et al., 2021; Joss et al., 2016). Another strength to optimize validity was the involvement of public collaborators when discussing search strategy, data extraction approach and terminology. Like for all kinds of literature studies, the risk of validity threats due to missing relevant papers is almost inevitable, and may be due to variations in terminology, quality of reporting PPI as well as variation of indexation across databases. The consequences of missing papers for the interpretation of overall findings are in principle uncertain however in our opinion outperformed by the study strengths.

## Conclusion

This scoping review revealed a diversity of how PPI was carried out, which mirrors the varied nature of OT health research and underline the need for a flexible PPI approach. To verify PPI as an integral part of the entire research process, PPI activities and evaluation of the impact should be documented in future research. We suggest a higher level of comprehensiveness and consistency in the reporting of PPI, which may be accomplished using reporting checklists. Submission guidelines for reporting PPI in scientific journals support the progress of PPI. OT researchers may contribute to the further evolvement of PPI by initiating, evaluating, and reporting PPI based on the aligning principles of involvement and participation in OT and PPI.

## Supplemental Material

sj-docx-1-otj-10.1177_15394492221096058 – Supplemental material for Patient and Public Involvement in Occupational Therapy Health Research: A Scoping Review Click here for additional data file. Supplemental material, sj-docx-1-otj-10.1177_15394492221096058 for Patient and Public Involvement in Occupational Therapy Health Research: A Scoping Review by Toril Beate Røssvoll, Tove Aminda Hanssen, Jan H. Rosenvinge, Kristin Liabo and Gunn Pettersen in OTJR: Occupation, Participation and Health

sj-docx-2-otj-10.1177_15394492221096058 – Supplemental material for Patient and Public Involvement in Occupational Therapy Health Research: A Scoping Review Click here for additional data file. Supplemental material, sj-docx-2-otj-10.1177_15394492221096058 for Patient and Public Involvement in Occupational Therapy Health Research: A Scoping Review by Toril Beate Røssvoll, Tove Aminda Hanssen, Jan H. Rosenvinge, Kristin Liabo and Gunn Pettersen in OTJR: Occupation, Participation and Health
