# Supplementary material for: Patient and Public Involvement in Occupational Therapy Health Research: A Scoping Review
Source: OTJR (Thorofare N J). 2022 May 13;43(1):119–26. doi: 10.1177/15394492221096058 (PMC9729968; doi:10.1177/15394492221096058)
Supplement: sj-docx-1-otj-10.1177_15394492221096058 – Supplemental material for Patient and Public Involvement in Occupational Therapy Health Research: A Scoping Review [file sj-docx-1-otj-10.1177_15394492221096058.docx]

**Supplement 1**

*Search Terms Used*

|  | MEDLINE (Ovid) |  | CINAHL (EBSCOhost) |  | Scopus | Embase (Ovid) |
| --- | --- | --- | --- | --- | --- | --- |
| 1 | consumer participation.ab,kf,ti. or exp Community Participation/ | 1 | MH (“consumer participation”) | 1 | “consumer participation” | Consumer participation.ab,kw,ti. |
|  |  | 2 | TI “consumer participation” or  AB “consumer participation” |  |  |  |
| 2 | patient participation.ti,ab,kw | 3 | TI “patient participation” or AB “patient participation” | 2 | “patient participation” | Patient participation/ |
| 3 | 1 or 2 | 4 | 1 or 2 or 3 | 3 | 1 or 2 | 1 or 2 |
| 4 | (patient* or public or lay or people or consumer* or user* or citizen*).ti,ab. | 5 | TI ( patient* or public or lay or people or consumer* or user* or citizen )  OR  AB ( patient* or public or lay or people or consumer* or user* or citizen ) | 4 | ( TITLE-ABS-KEY ( patient*  OR  public  OR  lay  OR  people  OR  consumer*  OR  user*  OR  citizen* ) | (patient* or public or lay or people or consumer* or user* or citizen*).ti,ab,kw. |
| 5 | (participat* or involv* or engag*).ti,ab. | 6 | TI ( participat* or involv* or engag* )  OR  AB ( participat* or involv* or engag* ) | 5 | TITLE-ABS-KEY ( participat*  OR  involv*  OR  engag* ) | (participat* or involv* or engag*).ti,ab,kw. |
| 6 | (Health or research).ti,ab. | 7 | TI (Health or research) or AB (Health or research) | 6 | TITLE-ABS-KEY ( health  OR  research ) | (health or research).ti,ab,kw. |
| 7 | 4 and 5 and 6 | 8 | 5 and 6 and 7 | 7 | 4 and 5 and 6 | 4 and 5 and 6 |
| 8 | (partners or partnership).ti,ab | 9 | TI ( partners or partnership )  OR  AB ( partners or partnership ) | 8 | TITLE-ABS-KEY ( partners  OR  partnership ) | (partners or partnership).ti,ab,kw. |
| 9 | 6 and 8 | 10 | 7 and 9 | 9 | 6 AND 8 | 6 and 8 |
| 10 | 3 or 7 or 9 | 11 | 4 or 8 or 10 | 10 | 3 or 7 or 9 (“#1 OR #3 OR #4) | 3 or 7 or 9 |
| 11 | Occupational therapy. ab,kf,ti. OR  Occupational therapy/ | 12 | (MH “occupational therapy+”) OR “occupational therapy” | 11 | “occupational therapy” | Occupational therapy/ or occupational therapy.ab,kw,ti. |
| 12 | Occupational Therapists/ or "occupational therapist*". ab,kf,ti. | 13 | (MH “occupational therapists”) | 12 | “occupational therapist*” | Occupational therapist/ or “occupational therapist”.ab,kw,ti. |
| 13 | Occupational science.ab,kf,ti. | 14 | (MH “occupational science”) OR “occupational science” | 13 | “occupational science” | Occupational science/ or “occupational science”.ab,kw,ti. |
| 14 | 12 or 13 or 14 | 15 | 12 or 13 or 14 | 14 | 11 or 12 or 13 | 12 or 13 or 14 |
| 15 | 10 AND 16 | 16 | 11 AND 15 | 15 | 10 and 14 / #5 and #6 | 10 and 15 |

Time limitation (all four databases): from 2010 to present.

Search performed in august 2020.
