# Supplementary material for: Patient and Public Involvement in Occupational Therapy Health Research: A Scoping Review
Source: OTJR (Thorofare N J). 2022 May 13;43(1):119–26. doi: 10.1177/15394492221096058 (PMC9729968; doi:10.1177/15394492221096058)
Supplement: sj-docx-2-otj-10.1177_15394492221096058 – Supplemental material for Patient and Public Involvement in Occupational Therapy Health Research: A Scoping Review [file sj-docx-2-otj-10.1177_15394492221096058.docx]

**Supplement 2**

*Data Charting Form*

| Title |  |
| --- | --- |
| Journal |  |
| Author |  |
| Year of publication |  |
| Country (authors) |  |
| Study aim(s) / objective |  |
| Study population |  |
| Design |  |
| The PPI terms used |  |
| PPI approach |  |
| Impacts of PPI described |  |
